# A discussion on the genus *Fomitiporella* (Hymenochaetaceae, Hymenochaetales) and first record of *F.americana* from southern South America

**DOI:** 10.3897/mycokeys.38.27310

**Published:** 2018-08-28

**Authors:** María Belén Pildain, Rodrigo Reinoso Cendoya, Beatriz Ortiz-Santana, José Becerra, Mario Rajchenberg

**Affiliations:** 1 Centro Forestal CIEFAP - CONICET, C.C. 14, 9200 Esquel, Chubut, Argentina; 2 Universidad Nacional de la Patagonia S.J. Bosco, Sede Esquel, Facultad de Ciencias Naturales, Ruta 259 km 14.6, 9200 Esquel, Chubut, Argentina; 3 Laboratorio de Química de Productos Naturales, Facultad de Ciencias Naturales y Oceanográficas, Universidad de Concepción, Concepción, Región del Bío Bío, Chile; 4 Center for Forest Mycology Research, US Forest Service, Northern Research Station, One Gifford Pinchot Drive, Madison, 53726, WI, USA; 5 Universidad Nacional de la Patagonia S.J. Bosco, Sede Esquel, Facultad de Ingeniería, Ruta 259 km 14.6, 9200 Esquel, Chubut, Argentina

**Keywords:** Hymenochaetaceae, phylogeny, taxonomy, wood-rotting fungi

## Abstract

*Fomitiporella* has traditionally been delimited based on the gross morphology of the basidiomes, hyphal structure and basdiospores. Recently, phylogenetic studies supported the incorporation of an extensive number of species within the genus. Although most of its species are nested in the ‘Phellinotus clade’ (Hymenochaetaceae, Basidiomycota), genera such as *Arambarria*, *Inocutis* and *Phellinotus* were not included in previous analysis. To further our understanding of the genus, new sequences from 28S and ITS nuc rDNA genes were jointly analysed with a large selection of taxa in the ‘Phellinotus clade’, also with re-examination of morphological and ecological data. Results showed several lineages in what has hitherto been considered to represent *Fomitiporella*, indicating that the genus is paraphyletic as presently circumscribed. There is a well-supported *Fomitiporella* core group that includes the type species and nine other monophyletic lineages with high support, of which those representing *Arambarria*, *Inocutis* and *Phellinotus* are distinct from the *Fomitiporella* core group by macro and micromorphological traits and/or biogeographic distribution. *Fomitiporellaamericana*, a species described from SE USA, was found in the Patagonian forests of southern Argentina and Chile; it is the taxon responsible for the white heart-rot found on standing *Austrocedruschilensis* and one of the taxa decaying wooden tiles of historic churches in Chiloé Is., Chile.

## Introduction

*Fomitiporella* Murrill [type species *F.umbrinella* (Bres.) Murrill] was originally described to encompass poroid Hymenochaetaceae (Hymenochaetales, Basidiomycota) with resupinate and perennial basidiome that present a thin context, ovoid to globose basidiospores with brown walls and lacking setae of any sort (Murrill 1907). As the species present a dimitic hyphal system, the genus was, for many years, considered a synonym of *Phellinus* Quél. (Ryvarden and Johansen 1980, Larsen and Cobb-Poulle 1990, Ryvarden 1991, Ryvarden and Gilbertson 1994, Dai 1999, Núñez and Ryvarden 2000, amongst others). Nevertheless, the genus received molecular support by Wagner and Fischer (2002) through the comparison and analyses of 28S DNA markers, a fact later on confirmed by Zhou (2014). Ji et al. (2017, 2018) broadened the concept of *Fomitiporella* on the basis of a wide sampling of specimens and species from Central America, USA, Europe, China and Vietnam, studies based on morphological examinations and separated phylogenetic analyses based on nuc rDNA ITS and 28S data sets. Their studies, though, did neither incorporate nor discuss the positions of several genera described previously, namely *Arambarria* Rajchenb. & Pildain, *Inocutis* Fiasson & Niemelä and *Phellinotus* Drechsler-Santos, Robledo & Rajchenb., published in works that showed the complex relations within the members of “Phellinotus clade”, where *Fomitiporella* is included (Wagner and Fisher 2002, Rajchenberg et al. 2015, Drechsler-Santos et al. 2016, Pildain et al. 2017).

**Figure 1.**
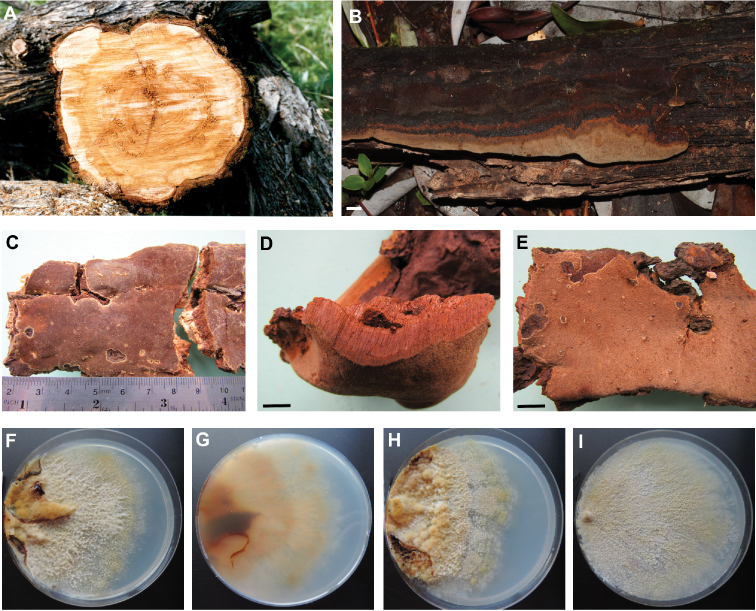
*Fomitiporellaamericana*, damage and morphology. **A** White heart-rot caused by the fungus in a section of a felled *Austrocedruschilensis***B–E** Basidiomes **B** Specimen RDS 1768 (=MR 12602, Chile) **C, E** Specimen MR 10946 (Chile) **D** Specimen MR 12060 (Argentina) **F–I** Macroscopic features of cultures **F** Strain CIEFAPcc 88, frontal view **G** Strain CIEFAPcc 88, reverse view **H** Strain CIEFAPcc 516 **I** Strain CIEFAPcc 595. Scale bar = 10 mm. Petri dishes measure 9 cm in diameter.

In Patagonia, Argentina, the native Cordilleran cypress [*Austrocedruschilensis* (D. Don) Pic. Sern. & Bizzarri, Cupressaceae] has been the subject of continuous research regarding the fungus responsible for the white heart-rot (WHR) present in living trees (Figure 1A). Studies on the wood-rots (Barroetaveña and Rajchenberg 1996) and search of the associated wood-rotting mycobiota (Rajchenberg 2002) were produced but were unsuccessful in identifying the WHR causing agent. Rajchenberg et al. (2015) included strains of this WHR fungus in their phylogenetic study of poroid Hymenochaetaceae from Patagonia. These strains clustered in a group of species that included *Fomitiporellacaryophylli* (Racib.) T. Wagner & M. Fischer (a strain from India, Wagner and Fischer 2002) and *Fulvifomesinermis* (Ellis & Everh.) Y.C. Dai (strains from China, Zhou 2014), but the species remained unnamed. In the last years, the search of poroid Hymenochaetaceae (Hymenochaetales, Basidiomycota) from southern Chile allowed us to find specimens that turned out to match the purported species.

The aims of this work were (1) to produce separated and combined phylogenetic analyses based on ITS and 28S markers of *Fomitiporella* in order to discuss its phylogenetic relationships and (2) to record *Fomitiporellaamericana* from southern South America.

## Methods

**Study areas.** Specimens of poroid Hymenochaetaceae were collected in the Valdivian Rainforest and the Subtropical Xerophytic and Durifoliated Forests of southern Chile (Hueck 1978, Donoso Zegers 1993) and in the Patagonian Andes forests of continental Argentina (Cabrera 1971).

**Specimens and cultures.** Specimens were dried and preserved in the Phytopathological Herbarium, Centro Forestal CIEFAP at the senior author’s address. See Suppl. material 1: Table S1 for specimens’ data. Many specimens determined as *Phellinusinermis* (Ellis & Everh.) G. Cunn. (Rajchenberg 2006) [present name *Fomitiporellainermis* (Ellis & Everh.) Murrill] from these areas were incorporated in this study.

Cultures were isolated by placing small portions of contextual tissue of basidiome and/or small portions of the associated wood-rot in the substrate in 2% malt extract agar medium. Morphological features of cultures (Nobles 1965, Stalpers 1978) were used to corroborate their affiliation to Hymenochaetaceae. Strains were deposited at the Culture Collection, Centro Forestal CIEFAP at the senior author’s address. Cultures of related specimens were included in the study. Strains isolated from the white heart-rot found in standing *Austrocedruschilensis* in Patagonia and previously determined as Hymenochaetaceae sp. (Barroetaveña and Rajchenberg 1996, Rajchenberg et al. 2015) were also incorporated, as well as a new strain of *F.inermis* from the USA. See Suppl. material 1: Table S1 for strains’ data.

**DNA extraction and PCR conditions.** DNA was extracted from basidiomes or freshly collected mycelium from pure culture grown in liquid malt peptone broth with 10% (v/v) of malt extract (Merck) and 0.1% (w/v) Bacto peptone (Difco), in 15 ml tubes at 24 °C in the dark. DNA extractions were carried out with the UltraCleanTM Microbial DNA Isolation Kit (MO BIO Laboratories Inc., Solana Beach, California), following the manufacturers’ protocols. PCR for the partial 28S (LSU gene that includes the D1/D2 domains) was performed with the primer pairs LROR-LR5 (Vilgalys and Hester 1990) and the full Internal Transcribed Spacer region (i.e. ITS1, ITS2 and the intervening 5.8S RNA gene; further referred as ITS) with ITS5-ITS4 (White et al 1990). The PCR conditions were described in Rajchenberg et al. (2015): 95 °C for 2 min, 30 cycles of 94 °C for 30 s, 52 °C for 30 s, 72 °C for 1 min, followed by 72 °C for 8 min. The amplified fragments were purified and sequenced at the DNA Synthesis and Sequencing Facility, Macrogen (Seoul, Korea). Sequences generated in this study were submitted to GenBank (cf. Suppl. material 1: Table S1).

**Sequence and phylogenetic analyses.** Obtained sequences were blasted against the nucleotide database from Genbank (https://blast.ncbi.nlm.nih.gov/Blast.cgi). Available ITS and 28S sequences of the genera *Fomitiporella* obtained by Ji et al. (2017) were included. We also included the sequences of *Arambarria*, *Phellinotus*, *Inocutis* and *Phylloporia*. Sequences of *Fomitiporiapunctata* MUCL34101 and *Phellinusuncisetus* MUCL46231 were used as outgroups. Suppl. material 2: Table S2 lists the specimens used and their Genbank accession numbers.

Two datasets were analysed for this study: one for the ITS region and one for the 28S gene. Nucleotide sequences for the ITS region and 28S gene were initially edited with BioEdit 7.0.9.0 (Hall 1999), then aligned using L-INS-i strategy as implemented in MAFFT v 7.0 (Katoh and Standley 2013) and manually adjusted using MEGA version 6 (Tamura et al. 2013). Ambiguously aligned regions were eliminated using Gblocks 0.91b (Castresana 2000). The final ITS dataset comprised 53 sequences and 602 characters including gaps and the LSU dataset comprised 47 sequences and 882 characters including gaps. The datasets were combined for concatenated analyses using Mequite 3.40 (Maddison and Maddison 2018). The best-fit models of evolution were determined using the AIC criterion (Akaike 1974), implemented in jModelTest (Posada 2008, http://darwin.uvigo.es) and were HKY+G and TrN+I+G for ITS and 28S respectively. Phylogenetic analysis of the individual and combined dataset was performed using maximum likelihood (ML) and Bayesian Analyses (BA) optimality criteria. The ITS and LSU partitions included 599 and 881 characters, respectively, for a combined data matrix of 1480 characters. The number of included taxa were 44 for both ITS and LSU. Branch support was determined using nonparametric bootstrapping implemented in RAxML 7.2.8 (Stamatakis et al. 2014), using the default parameters, executed on the CIPRES (Cyberinfrastructure for Phylogenetic Research) Science Gateway V. 3.1 (http://www.phylo.org/sub_sections/portal/, Miller et al. 2010) with bootstrap support values calculated with 1000 repetitions. Bayesian phylogenetic analyses were performed using Mr Bayes v. 3.2.2 (Ronquist and Huelsenbeck 2003) for 8,000,000 generations, with four chains and trees sampled every 100 generations. The first 80,000 generations were discarded as the burn-in. Log files for each run were viewed in Tracer v1.6.0 (http://evolve.zoo.ox.ac.uk/software.html/tracer/) to determine convergence. Branch support was assessed using posterior probabilities calculated from the posterior set of trees after stationarity was reached. Trees generated prior to stationarity were discarded and the rest of the trees were summarised in a majority-rule consensus tree from the four independent runs. Alignments have been deposited at TreeBase: http://purl.org/phylo/treebase/phylows/study/22728.

## Results

### Phylogeny

**Figure 2.**
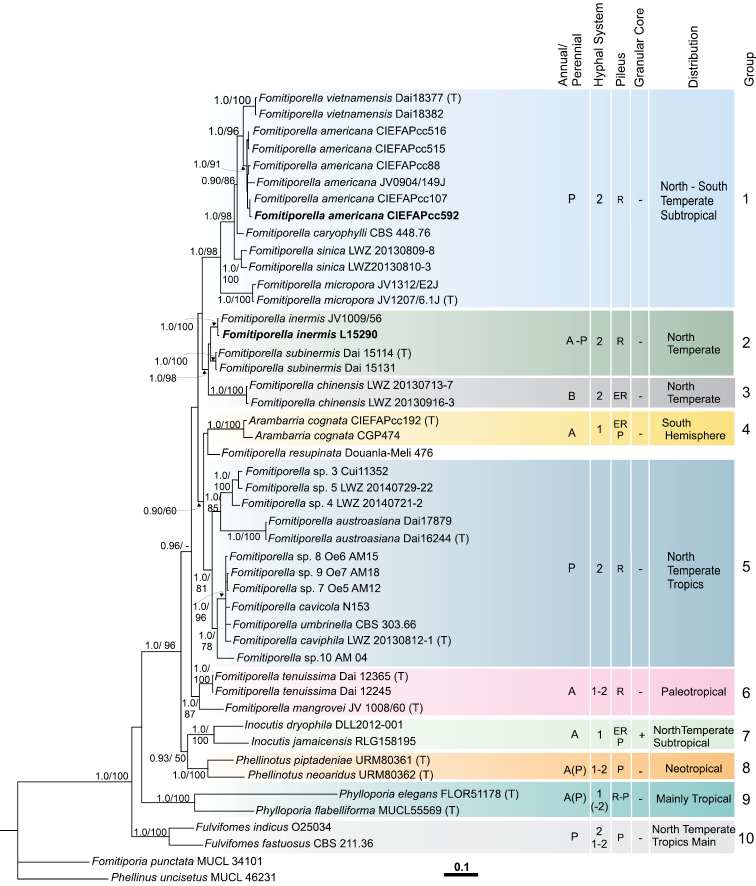
Phylogram generated from nuc rDNA ITS+28S combined sequence data with Bayesian and maximum likelihood analysis. Maximum likelihood (ML) bootstraps from 1000 iterations. Bayesian posterior probabilities (BPP) from 1000 iterations (8 million runs sampling every 100th iteration). Bootstrap values ≥ 50% (ML) followed by the Bayesian posterior probability (≥ 90%) are indicated in the node branches; -: support values lower than 50/90%. Bold type identifies new obtained sequences. T indicates sequences obtained from the genic type species. Horizontal coloured stripes distinguish different clades as treated in the text. Horizontal stripes point out morphological and distributional features of taxa. (A) annual basidiome. (P) perennial basidiome. (1) Monomitic hyphal system. (2) Dimitic hyphal system. (ER) effused reflexed. (P) pileate. (R) resupinate. (-) granular core absent. (+) granular core present.

Two loci analyses of 45 taxa inferred from Bayesian analyses (BA) and Maximum likehood (ML) were performed. The phylogenetic analyses included the simple and combined ITS + 28S concatenated dataset (Figure 2). Combined ITS and 28S analyses confirmed that members of *Fulvifomes*, *Phylloporia*, *Phellinotus*, *Arambarria*, *Inocutis* and *Fomitiporella* are closely related and form a strong monophyletic group (BA 1.0, ML 100) named “Phellinotus clade” by Dreschler et al. (2015). *Fulvifomes* and *Phylloporia* occupy a more basal position and form highly supported subclades. *Phellinotus*, *Arambarria*, *Inocutis* and *Fomitiporella* taxa clustered together as a monophyletic clade. Within this group, five well defined groups were observed in the combined ITS and 28S concatenated analyses (Fig. 1):

(1) *Inocutis*, *Phellinotus* (BA 0.93, ML 50); (2) *Fomitiporellatenuissima* is closely related to *F.mangrovei* but the relationships with the remaining species were not clear (BA 1.0, ML 98); (3) *Arambarria*, *Fomitiporellaaustroasiana*, *F.cavicola*, *F.caviphila*, *F.resupinata*, *F.umbrinella* (BA 0.9, ML 60); (4) *F.inermis*, *F.subinermis*, *F.chinensis* (BA 1.0, ML 98); and (5) *Fomitiporellamicropora*, *F.sinica*, *F.caryophylli*, *F.americana*, *F.vietnamensis*, (BA 1.0, ML 98) . The genus *Fomitiporella*, as currently defined, is paraphyletic, with the type species as part of the clade *Arambarria*, *Fomitiporellaaustroasiana*, *F.cavicola*, *F.resupinata*, *F.umbrinella* and the additional lineages occurring in the core “Phellinotus clade”; whereas *Fomitiporellasinica*, *F.americana*, *F.caryophylli*, *F.micropora* (BA 1.0, ML 98) may not be closely related to the *Fomitiporella* core group where the type (*F.umbrinella*) is included.

The internal topology of the “Phellinotus clade” is much better resolved in ITS + 28S than in the single gene datasets, with more than 85% of the nodes receiving strong support (ITS: 75%, Suppl. material 3: Figure S1; 28S: 65%, Suppl. material 4: Figure S2). Within these conflicts that had moderate support (BA <0.90 and/or ML <75%) appears the group formed by *Inocutis* and *Phellinotus* (group not supported in the 28S phylogenetic analysis; cf. Suppl. file S4) and *Arambarria*, *Fomitiporellacavicola*, *F.resupinata*, *F.umbrinella* and *F.tenuissima* with their uncertain position, while *F.americana* appears as a species with north and southern hemisphere members (see below).

Patagonian sequences (CIEFAP515, CIEFAP516, CIEFAP592) of the white heart-rot fungus, responsible for *A.chilensis* decay, fell within *Fomitiporellaamericana*, a species recently described from SE USA (Ji et al. 2017) (BA 1.0/0.9/0.99, ML 91/70/60 combined ITS + 28S; 28S and ITS, respectively) and differentiated from *Fomitiporellainermis* (formerly *Phellinusinermis*) (Figure 2, Suppl. materials 3, 4: Figures S1, S2). The closest sister group of *F.americana* is *F.vietnamensis* and together with *F.sinica* and *F.caryophylli* formed a strongly supported clade, where *F.micropora* is also included in a basal position (BA 1.0, ML 98 combined ITS + 28S). From the ITS phylogeny, it is noted that another strain that clustered with *F.americana* was the strain ‘Achao 50’ isolated from roof tiles of an historic wooden church from Chiloé Is. in southern Chile (Ortiz et al. 2014) (Suppl. material 3: Figure S1). Additionally from the ITS phylogeny, it could be observed that strains of *Fomitiporella* sp. (recorded as Hymenochaetales 1, 2 and 4 at GenBank), recorded by Cloete et al. (2015) and isolated from South African grapevines wood-rots associated with esca disease, did not match any known species and represent independent taxa (Suppl. material 3: Figure S1). From ‘Achao 50’ and Hymenochaetales 1, 2 and 4, there are only ITS sequences available and therefore only included in the ITS analysis.

The newly sequenced strain (L-15290) of *Phellinusinermis* sensu stricto from USA grouped with the other known sequence of *F.inermis* (Ji et al. 2017).

### Morphology, ecology and pathogenicity

Specimens previously determined as *Phellinusinermis* (Ellis & Everh.) G. Cunn. (Espinosa 1917, Rajchenberg 1987, 1995, 2006) from southern Argentina and Chile turned out to perfectly match phylogenetically with *Fomitiporellaamericana* Y.C. Dai, X.H. Ji & Vlasák . They are characterised by resupinate, perennial, flattened to pulvinate basidiomes that may also develop nodulose structures, reaching up to 1.3 mm thick (Figures 1B–E). They present a chestnut, chocolate brown to umbrinous hymenial surface with margins that are lighter in colour and, sometimes, receding. Pores vary from 4.5–7 mm, exceptionally smaller up to 8 mm. Hyphal system is dimitic. Basidiospores are ellipsoid to broadly ellipsoid, always with a straight, ventral, inner side, thick-walled with walls yellowish in water but dark chestnut in 5% KOH, IKI–, 4.5-5.5(6) × 3.5–4.5 µm. Spore size variability was shown by Rajchenberg (1995). Cultures showed macroscopical variation but were otherwise typical of the Hymenochaetaceae (Figures 1F–I) and as previously described by Barroetaveña and Rajchenberg (1996) under Hymenochaetaceae sp. For specimens examined, see Suppl. material 1: Table S1. Table 1 compares specimens from Patagonia with morphologically similar species described from USA and China.

In southern South America, *Fomitiporellaamericana* has a wide spectrum of hosts that includes living *Austrocedruschilensis* (Cupressaceae) and dead *Maytenusboaria* (Celastraceae), *Cryptocaryaalba* (Lauraceae), *Nothofagusdombeyi* and *N.nitida* (Nothofagaceae), *Diosteajuncea* (Verbenaceae), *Escallonia* sp. (Escalloniaceae), *Eucryphiacordifolia* and *Weinmanniatrichosperma* (Cunoniaceae), *Peumusboldus* (Monimiaceae), *Lumaapiculata* and *Tepualiastipularis* (Myrtaceae). It decays fallen trunks and branches but is also pathogenic to standing *A.chilensis*, being responsible for the WHR that has been recorded many years ago (Barroetaveña and Rajchenberg 1996).

## Discussion

This study incorporated for the first time all molecular information available for *Fomitiporella* species and related organisms pertaining to the ‘Phellinotus clade’ (Drechsler-Santos et al. 2016). Our combined analyses showed that *Fomitiporella*is paraphyletic as presently circumscribed by Ji et al. (2017, 2018), most notably the unresolved relationships with the well-recognised genera *Inocutis* (Fiasson and Niemelä 1984, Wagner and Fischer 2002), *Phellinotus* and *Arambarria*, all of which present differences in their gross morphology and in the nature of the hyphal structure. Amongst the poroid Hymenochaetaceae, *Inocutis* is distinguished morphologically by a combination of the monomitic hyphal system and the formation of a granular core in the context. It is associated with *Phellinotus* Drechsler-Santos, Robledo & Rajchenb. (Drechsler-Santos et al. 2016), that is distinguished by a monomitic context, dimitic trama of the tubes and by variable presence of a granular core in context (i.e. variably present in *Phellinotusneoaridus* Drechsler-Santos & Robledo). *Arambarria* Rajchenb. & Pildain (Rajchenberg et al. 2015, Pildain et al. 2017) appeared distant from the former and is monomitic throughout the basidiome and lacks a granular core in context. The three genera contrast with the fully dimitic *Fomitiporella* species.

In view of the molecular data presented here, *Fomitiporella* is paraphyletic and the treatment of the genus *Fomitiporella* by Ji et al. (2017, 2018) is artificial. In addition, since the combined phylogenetic analyses retrieved 10 monophyletic lineages with high support within the “Phellinotus clade”, it is possible to imagine different taxonomic scenarios:

1) To accept 10 different genera within the group: *Fulvifomes*; *Phylloporia*; *Inocutis*; *Phellinotus*; *Arambarria*; *Fomitiporella* (including *F.umbrinella*, *F.cavicola*, *F.austroasiana* and *Fomitiporella* spp. 3, 4, 5, 7, 8, 9 and 10); and the following taxa as 4 new genera: *Fomitiporellainermis* and *F.subinermis*; *F.chinensis*; *F.tenuissima* and *F.mangrovei*; and subclade *F.sinica*, *F.caryophylli*, *F.americana*, *F.vietnamensis* and *F.micropora*.

The problem with this solution is that a new genus for *F.chinensis* would only include, for the time being, one species.

The case of *F.resupinata* is unclear as it presents an uncertain position close to *Arambarria*. More materials from Africa are needed in order to ascertain its phylogenetic position.

Option ‘1’ would be the easiest solution from an ‘operational’ point of view, with statistical support comparable to those shown for recent genera that have been treated in the Hymenochaetaceae and accepted phylogenetically: *Onnia* (Ji et al. 2017), *Phellinidium* and *Coniferiporia* (Zhou et al. 2016) and *Neomensularia* (Wu et al. 2016), only to give some examples.

2) To group *Inocutis*, *Phellinotus*, *Arambarria* and *Fomitiporella* s.l. (Ji et al. 2017, 2018) as a unique genus *Fomitiporella*.

This option presents the following problems regarding:

Morphology: the genus would include full monomitic species (those included in *Inocutis* and *Arambarria*), full dimitic species (*Fomitiporella* s.l.) and species with monomitic context and dimitic trama of tubes (*Phellinotus*).

Taxonomy: the genus would encompass three well-established and recognised genera such as *Inocutis*, *Arambarria* and *Phellinotus*.

3) To maintain *Inocutis* and *Phellinotus* as independent genera and to group *Arambarria* under *Fomitiporella* s.l.

This option appears to be convenient but seems not consistent on the basis of the variable phylogenetic supports (Fig. 1, Suppl. files S3, S4) and, morphologically, because of the monomitic hyphal system of *Arambarria*.

Phylogenetic studies showed that specimens, previously recorded as *Phellinusinermis* from southern Argentina and Chile, match *Fomitiporellaamericana*, a taxon recently described from SE USA (Ji et al. 2017). The species is morphologically similar to *Fomitiporellainermis* and other taxa described from China that were previously recorded as *Fulvifomesinermis* (Ellis & Everh.) Y.C. Dai (Dai 2010). These species are presently accepted as *F.sinica* Y.C. Dai, X.H. Ji & Vlasák and *F.subinermis* Y.C. Dai, X.H. Ji & Vlasák (Ji et al. 2017). The prominent features of these species are shown in Table 1, which underlines that morphological differences between them are subtle, constituting a species complex. From a phylogenetic point of view, *F.americana* comes close to *F.sinica* and *F.caryophylli*, but distant from *F.inermis*, which grouped with *F.subinermis* and *F.chinensis* (Figure 2, ITS+28S). This shows that morphology has been conservative throughout the evolution of this group and is a limited criterion to distinguish taxa.

*Fomitiporellaamericana* was originally recorded on *Quercus* sp. but this study shows it is widely distributed on many hosts in southern South America. Our results also show that *F.americana* is one of the wood-rotting agents decaying historic wooden churches in Chiloé Is., southern Chile, recorded as isolate ‘Achao50’ (Ortiz et al. 2014). During many years, the causing agent of the WHR present in *A.chilensis* standing trees was unknown despite several efforts made to find it (cf. Introduction). Though many basidiomes of *F.americana* (as *Phellinusinermis*) had been found in the past, they never fruited on *A.chilensis*. For this reason, the match between isolates of the WHR fungus and the several materials from S Chile and Argentina came as a surprise, indicating the role of this species in the decay of standing trees. This study shows that *F.americana* appears to have a wide distribution in the Americas, for the moment apparently a species with an amphitropical distribution (i.e. present in temperate to cold temperate areas of the North and South Hemisphere). Whether it is also present in tropical areas needs to be verified; specimens determined as *inermis* have been recorded from Central Argentina (Robledo and Urcelay 2009), but their identity needs to be worked out from a phylogenetic point of view. To date, *F.americana* or any morphological similar taxon has not been recorded from Neotropical areas by Ryvarden (2004). Specimens of *P.inermis* recorded from New Zealand (Cunningham 1965) might represent a different taxon due to its effused-reflexed basidiomes (Parmasto et al. 1980) though they are microscopically akin (Rajchenberg 1987).

Our study incorporated a second strain and sequence of *Fomitiporellainermis* sensu stricto (i.e. L-15290, cf. Figure 2 and Suppl. material 1: Table S1) that perfectly matched that of J. Vlásak 1009/56 gathered on *Ilexmucronata* (Ji et al. 2017). Both showed to be close to *F.chinensis* and *F.subinermis* and far from *F.americana* and *F.sinica*.

As an ending remark, we point out that, before proposing taxonomic inferences coherent with phylogenetic results, it seems cautious to wait till more taxa are sampled and more loci are incorporated into phylogenetic analyses, also including taxa around *F.chinensis* and *F.tenuissima*. Incorporation of more sequences from more taxa may certainly impact the phylogeny, as the resolution of the phylogeny of “Phellinotus clade” is low. Operational units (genera) shown by phylogenetic analyses are certainly correctly defined but, if one admits a large *Fomitiporella* genus, one has to admit that we are unable to understand what biological and morphological features are leading the evolution of this group of Hymenochaetaceae.

**Table 1.** Morphological comparison of *Fomitiporellaamericana* with similar species from different geographic areas.

	*Fomitiporellaamericana* (USA) (Ji et al. 2017)	*Fomitiporellaamericana* (S Argentina and Chile)	*Fomitiporellainermis* (USA) (Ji et al. 2017)	*Fomitiporellasubinermis* (China) (Ji et al. 2017)	*Fomitiporellasinica* (China) (Ji et al. 2017)
Pores/mm	5–6	4.5–7	5–7	6–7	6–8
Spores length (µm)	(3.5)4–4.5	4.5–5.5(6)	4.5–5(5.5)	(4)4.5–5(5.5)	4–4.5
Spores width (µm)	(2.5)3–3.5(4)	3.5–4.5	3.5–4(4.5)	3.5–4	3–3.5
Spores shape	subglobose*	ellipsoid to broadly ellipsoid	broadly ellipsoid	subglobose*	broadly ellipsoid to globose*
Ecology	fallen trunks (FT)	living trees (L), generally FT	FT	root of trees	L and FT
Hosts	*Quercus* sp. (D) (Fagaceae)	Numerous hosts, see text	*Ilexmucronata* (Aquifoliaceae). Several substrata fide Lowe (1966)	Unknown angiosperm	*Casuarina* sp. (L) (Casuarinaceae) *Melia* sp. (L) (Meliaceae) *Rhododendron* sp. (D) (Ericaceae)

*But no subglobose spore was drawn in Ji et al. (2017). FT= fallen trunk; L= living tree; D= dead; L= living.

## Supplementary materials

Supplementary material 1 Table S1 Explanation note: List of specimens and strains studied. MR= specimens collected by M. Rajchenberg. RDS= R. Drechsler-Santos. This is an open access article distributed under the terms of the Creative Commons Attribution License (CC BY 4.0), which permits unrestricted use, distribution, and reproduction in any medium, provided the original author and source are credited.María Belén Pildain, Rodrigo Reinoso Cendoya, Beatriz Ortiz-Santana, José Becerra, Mario Rajchenberg

Supplementary material 2 Table S2 Explanation note: List of taxa presented in the phylogenetic analyses with GenBank accession numbers for the ITS and 28S sequences. (T) indicates type specimen. In bold, new generated sequences. This is an open access article distributed under the terms of the Creative Commons Attribution License (CC BY 4.0), which permits unrestricted use, distribution, and reproduction in any medium, provided the original author and source are credited.María Belén Pildain, Rodrigo Reinoso Cendoya, Beatriz Ortiz-Santana, José Becerra, Mario Rajchenberg

Supplementary material 3 Figure S1 Explanation note: Phylogenetic relationships of 53 Hymenochaetaceae specimens inferred from nuc rDNA ITS sequences. Topology from Bayesian analysis (BA), Maximum likelihood (ML) bootstraps from 1000 iterations. Bayesian posterior probabilities (BPP) from 1000 iterations (8 million runs sampling every 100th iteration). Bootstrap values ≥ 50% (ML) followed by the Bayesian posterior probability (≥ 90%) are indicated in the node branches; -: support values lower than 50/90%. Bold type identifies new obtained sequences. T indicates sequences obtained from the genetic type species. This is an open access article distributed under the terms of the Creative Commons Attribution License (CC BY 4.0), which permits unrestricted use, distribution, and reproduction in any medium, provided the original author and source are credited.María Belén Pildain, Rodrigo Reinoso Cendoya, Beatriz Ortiz-Santana, José Becerra, Mario Rajchenberg

Supplementary material 4 Figure S2 Explanation note: Phylogenetic relationship between *Fomitiporella* and related genera inferred from the nuc rDNA 28S dataset. Topology from Bayesian analysis (BA), Maximum likelihood (ML) bootstraps from 1000 iterations. Bayesian posterior probabilities (BPP) from 1000 iterations (8 million runs sampling every 100th iteration). Bootstrap values ≥ 50% (ML) followed by the Bayesian posterior probability (≥ 90%) are indicated in the node branches; -: support values lower than 50/90%. Bold type identifies new obtained sequences. T indicates sequences obtained from the genetic type species. This is an open access article distributed under the terms of the Creative Commons Attribution License (CC BY 4.0), which permits unrestricted use, distribution, and reproduction in any medium, provided the original author and source are credited.María Belén Pildain, Rodrigo Reinoso Cendoya, Beatriz Ortiz-Santana, José Becerra, Mario Rajchenberg
